# Antibiotics ameliorate lupus-like symptoms in mice

**DOI:** 10.1038/s41598-017-14223-0

**Published:** 2017-10-20

**Authors:** Qinghui Mu, Vincent J. Tavella, Jay L. Kirby, Thomas E. Cecere, Matthias Chung, Jiyoung Lee, Song Li, S. Ansar Ahmed, Kristin Eden, Irving Coy Allen, Christopher M. Reilly, Xin M. Luo

**Affiliations:** 1 Department of Biomedical Sciences and Pathobiology, Virginia-Maryland College of Veterinary Medicine, Virginia Tech, Blacksburg, Virginia USA; 2 Department of Mathematics, Virginia Tech, Blacksburg, Virginia USA; 3 Department of Crop and Soil Sciences, Virginia Tech, Blacksburg, Virginia USA; 4 Edward Via College of Osteopathic Medicine, Blacksburg, Virginia USA

## Abstract

Gut microbiota and the immune system interact to maintain tissue homeostasis, but whether this interaction is involved in the pathogenesis of systemic lupus erythematosus (SLE) is unclear. Here we report that oral antibiotics given during active disease removed harmful bacteria from the gut microbiota and attenuated SLE-like disease in lupus-prone mice. Using MRL/lpr mice, we showed that antibiotics given after disease onset ameliorated systemic autoimmunity and kidney histopathology. They decreased IL-17-producing cells and increased the level of circulating IL-10. In addition, antibiotics removed *Lachnospiraceae* and increased the relative abundance of *Lactobacillus* spp., two groups of bacteria previously shown to be associated with deteriorated or improved symptoms in MRL/lpr mice, respectively. Moreover, we showed that the attenuated disease phenotype could be recapitulated with a single antibiotic vancomycin, which reshaped the gut microbiota and changed microbial functional pathways in a time-dependent manner. Furthermore, vancomycin treatment increased the barrier function of the intestinal epithelium, thus preventing the translocation of lipopolysaccharide, a cell wall component of Gram-negative *Proteobacteria* and known inducer of lupus in mice, into the circulation. These results suggest that mixed antibiotics or a single antibiotic vancomycin ameliorate SLE-like disease in MRL/lpr mice by changing the composition of gut microbiota.

## Introduction

Little is known on the role of gut microbiota in systemic lupus erythematosus (SLE)^1,2^. Our research team has described the dynamics of gut microbiota in a classical SLE mouse model MRL/Mp-*Fas* ^*lpr*^ (MRL/lpr)^3^. In female lupus mice we found marked depletion of Lactobacilli, and increase of Clostridial species (*Lachnospiraceae*) together with increased bacterial diversity compared to age-matched healthy controls. Importantly, treatments that improved lupus symptoms in lupus mice also restored gut colonization of *Lactobacillus* spp. and decreased that of *Lachnospiraceae*. This suggests that attenuation of lupus disease may be achieved by changes of gut microbiota, but experimental evidence is lacking. Notably, MRL/lpr mice raised under germ-free (GF) conditions exhibit similar disease course and severity as mice housed under conventional conditions^4^, indicating that complete removal of gut microbiota started early in life—including both pathogenic and beneficial microbes—does not attenuate lupus. However, it remains unknown whether and how the removal of gut microbiota after lupus onset would affect the disease. The removal of commensal bacteria post disease onset can be achieved with appropriate antibiotics^5^. This would be more clinically relevant than GF experiments as treatments are usually given after the appearance of clinical signs.

Antibiotics are known to improve symptoms in rheumatoid diseases. Dr. Thomas M. Brown (1906–1989), a renowned rheumatologist, had used antibiotics to successfully treat many patients with rheumatoid arthritis (RA). While no studies have been reported yet that associate antibiotic use to SLE (except rare cases of sun sensitivity with trimethoprim and sulfamethoxazole), several clinical trials have suggested that the use of antibiotics is associated with a clinically significant improvement in disease activity in RA without notable side effects^6^. In animal models, the removal of commensal bacteria by either GF housing or antibiotic treatment reduces the severity of RA-like disease^7,8^, although one study has shown exacerbation of collagen-induced arthritis with partial depletion of gut flora^9^. In lupus-prone mice, acidified water that reduced bacterial diversity in the gut delayed the onset of nephritis and decreased the level of circulating anti-nuclear antibodies^10^. Besides rheumatic diseases, antibiotics have been recently shown to affect the severity of another autoimmune disease type 1 diabetes^11–16^. Most of these studies suggest that antibiotics exacerbate type 1 diabetes.

Here, we showed that antibiotic treatment initiated post disease onset ameliorated lupus-like symptoms likely by decreasing IL-17-producing cells in the spleen and kidney and increasing circulating IL-10. Importantly, antibiotics given at active-disease stage significantly altered the composition of gut microbiota, and most notably, increased the relative abundance of *Lactobacillus* spp. while decreasing that of *Lachnospiraceae*. Moreover, we showed that vancomycin, a single antibiotic known to remove Clostridia (to which *Lachnospiraceae* belongs to) and enrich Lactobacilli in both human and mouse^17–20^, was able to recapitulate the attenuated disease phenotype seen with the mixed antibiotic treatment. Furthermore, mathematical and functional analyses of the microbiome revealed vancomycin-induced changes that were related to Gram-negative bacteria and lipopolysaccharide (LPS). Vancomycin decreased intestinal permeability and reduced the concentration of LPS, a known inducer of murine lupus^21–25^, in the circulation, thus attenuating lupus. Together, these results suggest that antibiotics may be beneficial as a treatment for lupus.

## Results

### Antibiotics given post disease onset attenuated lupus

**Figure 1:**
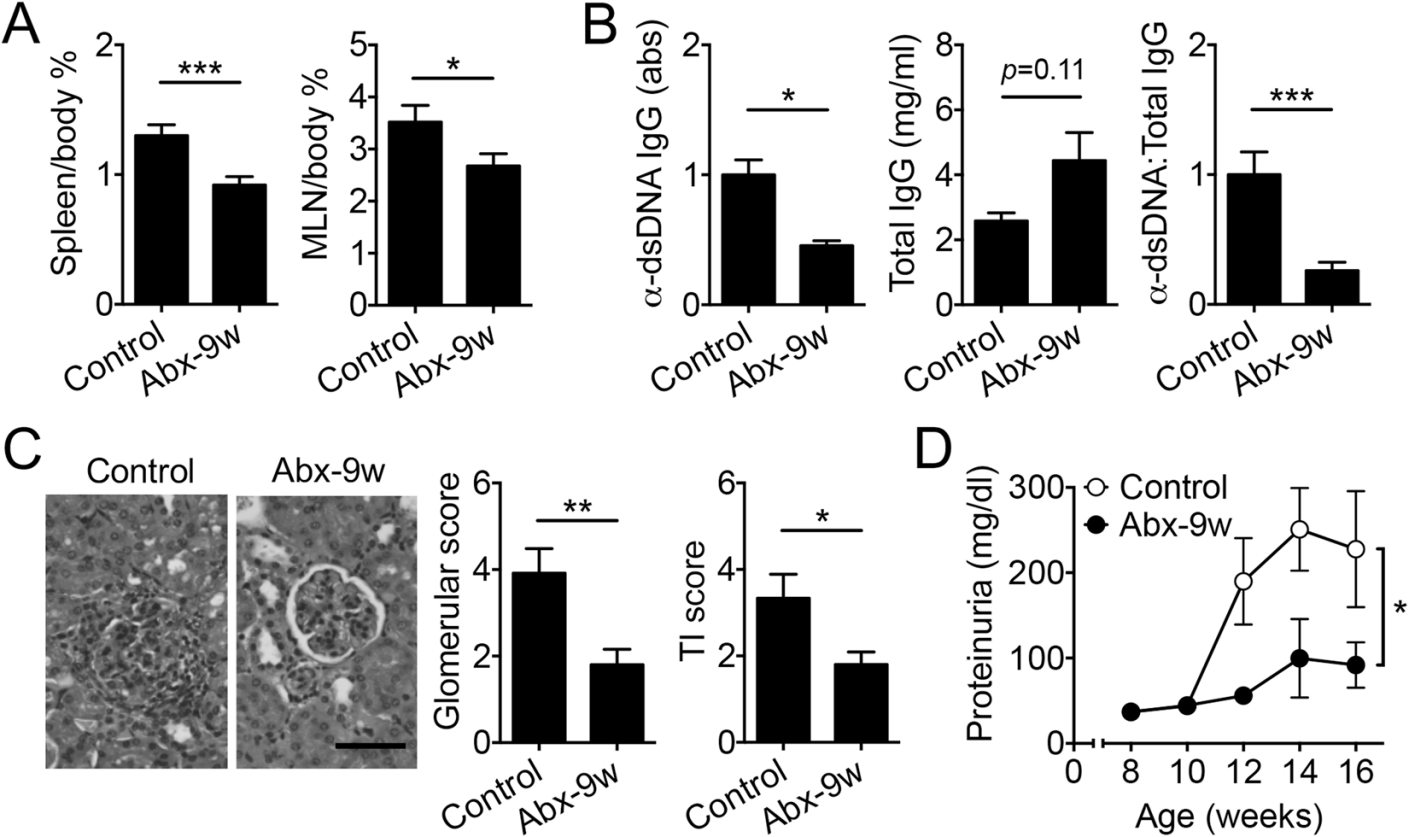
Antibiotic treatment started after lupus onset led to disease attenuation. (**A**) Spleen and MLN tissue weight to body weight ratio (%) at 16 weeks of age (n ≥ 8 per group). Control: no antibiotics. Abx-9w: oral antibiotics were given starting from 9 weeks of age and post disease onset. (**B**) Level of anti-dsDNA IgG in the mouse serum, total IgG, and the ratio of anti-dsDNA IgG to total IgG at 16 weeks of age (n ≥ 8 per group). (**C**) Renal histopathology at 16 weeks of age (n ≥ 8 per group). Left: representative PAS-stained kidney sections; bar equals 200 µm. Middle: glomerular score. Right: tubulointerstitial (TI) score. (**D**) Level of proteinuria over time (n ≥ 8 per group). *p < 0.05, **p < 0.01, ***p < 0.001.

The onset of autoimmune responses in female MRL/lpr mice is as early as 6 weeks of age^26^. To determine the effects of antibiotics on active disease in lupus-prone MRL/lpr mice, we treated female mice with a combination of antibiotics (ampicillin, neomycin, metronidazole and vancomycin^5^) started at 9 weeks of age and post disease onset. The treatment led to enlarged ceca as expected for antibiotic treatments, whereas the overall body weight did not change (data not shown). Spleen and mesenteric lymph node (MLN) weights were significantly decreased with antibiotic treatment compared to controls (Fig. 1A). The serum level of IgG autoantibodies against double-stranded (ds) DNA was also significantly reduced by the mixed antibiotic treatment (Fig. 1B). As kidney inflammation (or lupus nephritis) affects up to 60% of lupus patients^27,28^, we determined the renal function by measuring proteinuria and kidney histopathology. Both glomerular and tubulointerstitial scores were significantly decreased with antibiotic treatment compared to controls (Fig. 1C). The antibiotics also significantly reduced proteinuria (Fig. 1D), suggesting improvement of renal function. Together, these results indicate amelioration of lupus-like disease in female MRL/lpr mice with post-disease-onset antibiotic treatment.

**Figure 2:**
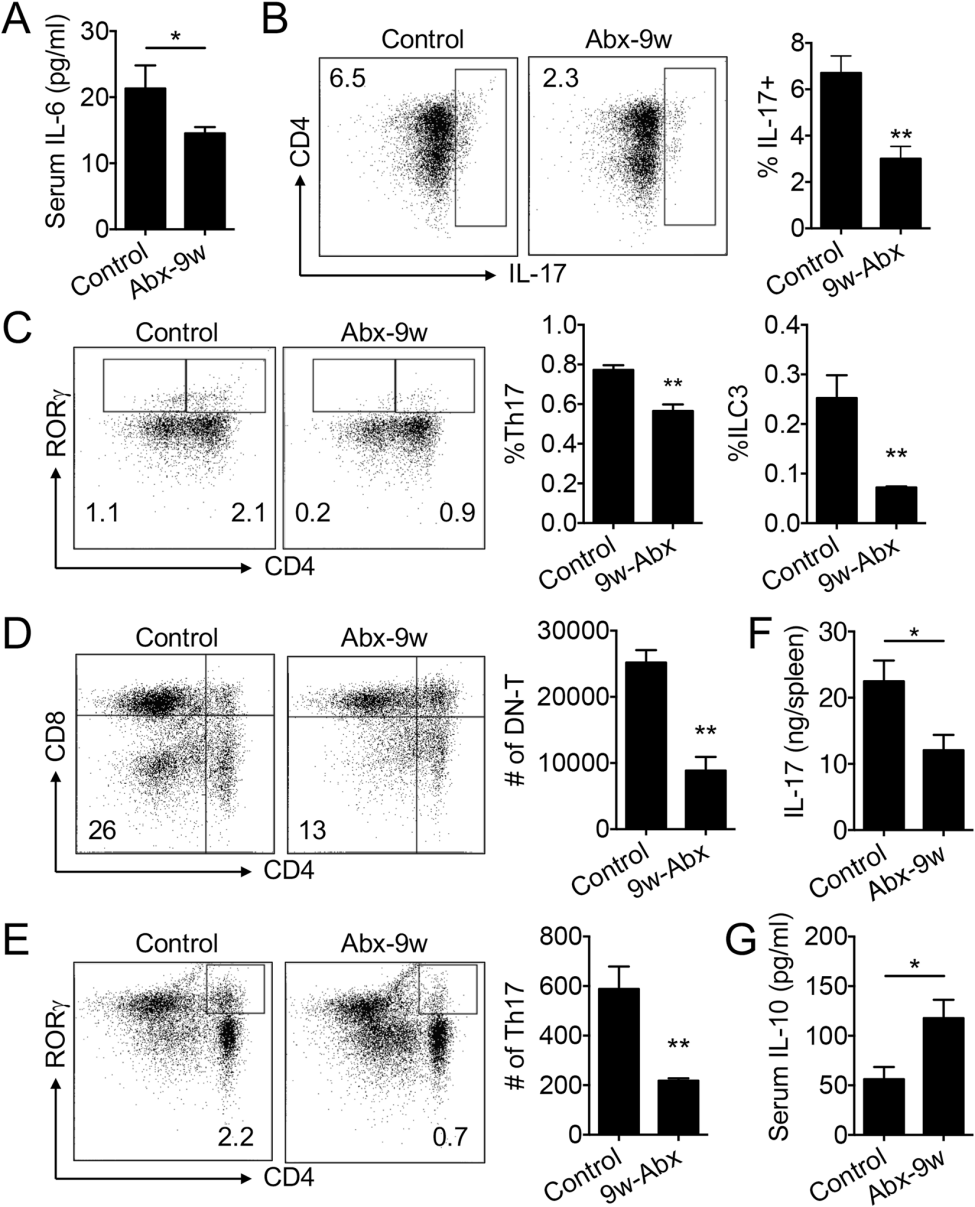
Antibiotic treatment decreased IL-17-producing cells in the spleen and kidney and increased IL-10 in the circulation. (**A**) Serum level of IL-6 at 16 weeks of age (n ≥ 7 per group). (**B**) Intracellular staining of IL-17 and the percentage of IL-17-producing cells in the spleen at 16 weeks of age (n = 4 per group). (**C**) FACS analysis of CD4^+^RORγ^+^ Th17 cells and CD4^−^RORγ^+^ ILC3 cells in the spleen at 16 weeks of age (n = 4 per group). (**D,E**) FACS analysis of DN-T cells (**D**) and Th17 cells (**E**) in the kidney at 16 weeks of age (n = 4 per group). (**F**) Protein level of IL-17 in the spleen at 16 weeks of age (n ≥ 7 per group). (**G**) Serum level of IL-10 in the mouse serum at 16 weeks of age (n ≥ 7 per group). *p < 0.05, **p < 0.01.

We next sought to understand the underlying mechanism of how antibiotic treatment ameliorated lupus in female MRL/lpr mice by examining immune cell differentiation and inflammatory mediator production (Fig. 2). IL-6, known to promote lupus disease in both human and mouse due to its ability to increase T-helper (Th)17 differentiation and IL-17 production^29,30^, was significantly decreased in the serum with antibiotic treatment (Fig. 2A). We then quantified IL-17-producing cells in the spleen and kidney by using flow cytometry. As anticipated, antibiotic treatment significantly reduced the percentages of IL-17^+^ cells (Fig. 2B), CD3^+^CD4^+^RORγ^+^ Th17 cells and Lin^−^CD4^−^RORγ^+^ group 3 innate lymphoid cells (ILC3s) (Fig. 2C). Both Th17 cells and ILC3s are known producers of IL-17 in the spleen^31^, and their decrease suggests systemic downregulation of IL-17 with antibiotic treatment. In the kidney, the double negative (DN) T cells and Th17 cells are major sources of IL-17^32^ and they both were significantly reduced with post-disease-onset antibiotic treatment (Fig. 2D and Fig. 2E, respectively). As IL-17 was too low to be detected in the circulation, we measured IL-17 protein in the splenic extract that reflected the systemic level of IL-17. Post-disease-onset antibiotic treatment significantly reduced the level of splenic IL-17 (Fig. 2F). These results suggest that antibiotics may attenuate lupus-like disease in female MRL/lpr mice by suppressing the production of IL-17 from Th17 cells and ILC3s in the spleen and from DN-T and Th17 cells in the kidney. Interestingly, we also found a significant increase in serum IL-10 with antibiotic treatment (Fig. 2G) that correlated with an increase in the number of IL-10-producing cells in the MLN (Fig. S1A). IL-10 is known as a protective cytokine in MRL/lpr mice^33^.

### Antibiotics given post disease onset reshaped gut microbiota

**Figure 3:**
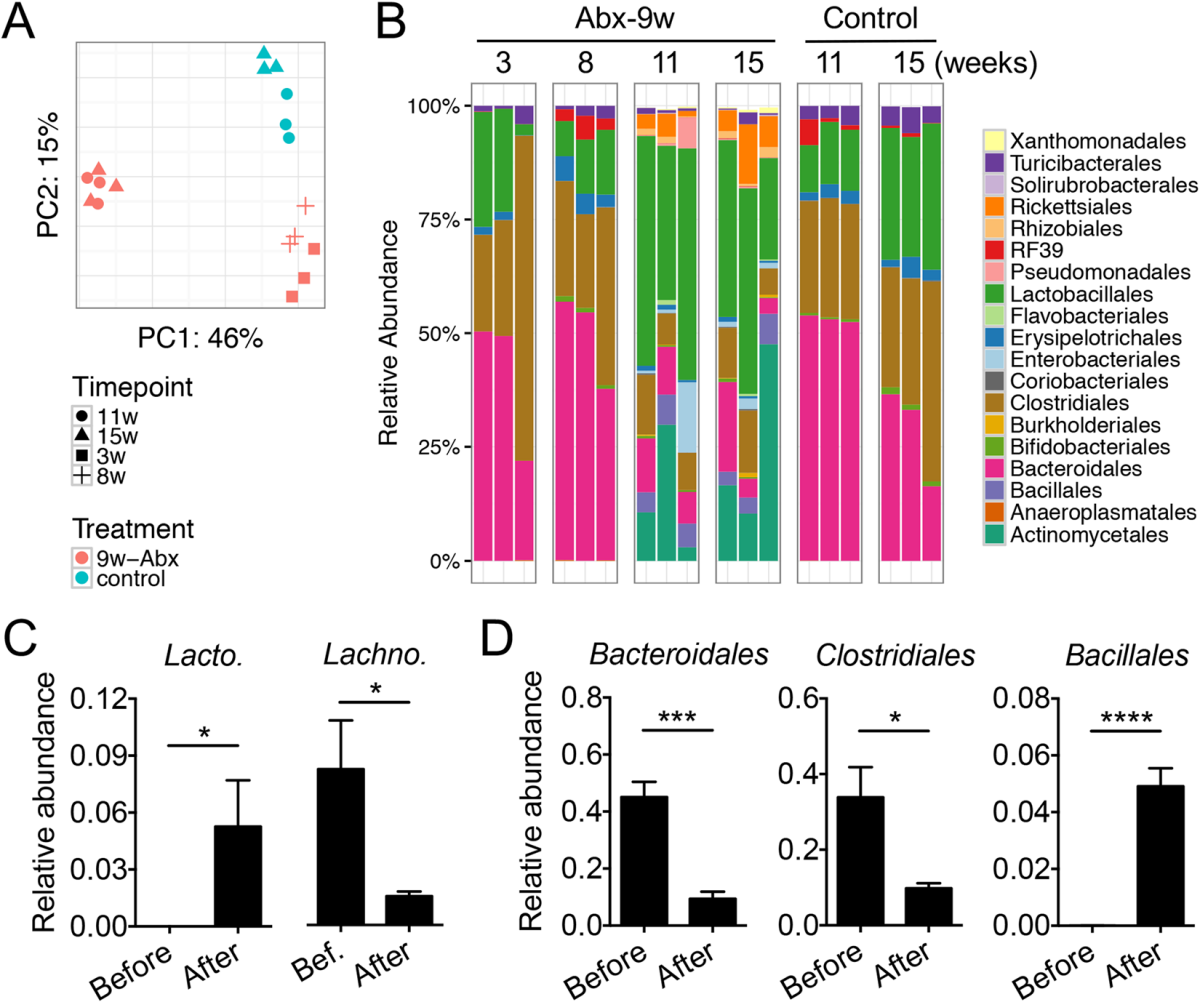
Composition of gut microbiota changed with antibiotic treatment. (**A**) Principal component analysis of fecal microbiota. w: weeks of age. *p* < 0.01, PERMANOVA. (**B**) Time-dependent changes of fecal microbiota. Bacterial taxa at the order level are shown. (**C**) Relative abundance of detectable *Lactobacillus* spp. (*Lacto*., left panel) and that of *Lachnospiraceae* (*Lachno*., right panel) (n = 6 per group). The detectable *Lactobacillus* spp. were *L. agilis*, *L. brevis*, *L. mucosae* and *L. reuteri*, and the sum of their relative abundance is shown. Before: 3 and 8 weeks of age. After: 11 and 15 weeks of age. (**D**) Relative abundance of *Bacteroidales*, *Clostridiales*, and *Bacillales* before and after antibiotic treatment initiated at 9 weeks of age (n = 6 per group). *p < 0.05, ***p < 0.001, ****p < 0.0001.

It has been recently reported that Th17 cells can migrate from the gut to the kidney to facilitate the development of lupus nephritis^34^, whereas the generation of Th17 cells in the gut is dependent on the gut microbiota^35^. We thus characterized the gut microbiota in antibiotics-treated mice. While antibiotic treatment initiated post disease onset did not decrease the bacterial diversity (Fisher index, Fig. S1B), it did reduce the bacterial load of fecal microbiota by 2 magnitudes (Fig. S1C). Based on 16 S rRNA sequencing analysis, the overall structure of the remaining gut bacteria was distinct from untreated animals (Fig. 3A; *p* < 0.01, PERMANOVA; red *vs*. blue symbols). In addition, the overall structure of the gut microbiota was different between the time points before (3 and 8 weeks of age) and after (11 and 15 weeks of age) antibiotic treatment initiation at 9 weeks of age (also in Fig. 3A; *p* < 0.01, PERMANOVA; comparison within the red symbols). Moreover, the antibiotic-treated group before given the treatment (3 and 8 weeks of age) shared similar gut microbiota composition with the Control group, but the antibiotic treatment initiated at 9 weeks of age appears to have altered the bacterial composition at 11 and 15 weeks of age (Fig. 3B). Further analysis on specific bacteria groups revealed that antibiotic treatment significantly increased the abundance of *Lactobacillus* spp. and significantly decreased the abundance of *Lachnospiraceae* (Fig. 3C), two groups of bacteria previously shown to be associated with improved or deteriorated symptoms in MRL/lpr mice, respectively^3^. *L. agilis*, *L. brevis*, *L. mucosae* and *L. reuteri*, in particular, were below detection limit before antibiotic treatment, whereas their abundance increased to about 5% after antibiotics-mediated enrichment. In addition to these changes, treatment with antibiotics removed significant amounts of *Bacteroidales* and *Clostridiales* while increasing the relative abundance of *Bacillales* (Fig. 3D). These results suggest that antibiotics given post disease onset reshaped the gut microbiota, removing potentially harmful bacteria (e.g., *Lachnospiraceae*) and enriching those that are associated with better disease outcomes (e.g., Lactobacilli).

### Vancomycin recapitulated the disease-attenuating effects of mixed antibiotics

**Figure 4:**
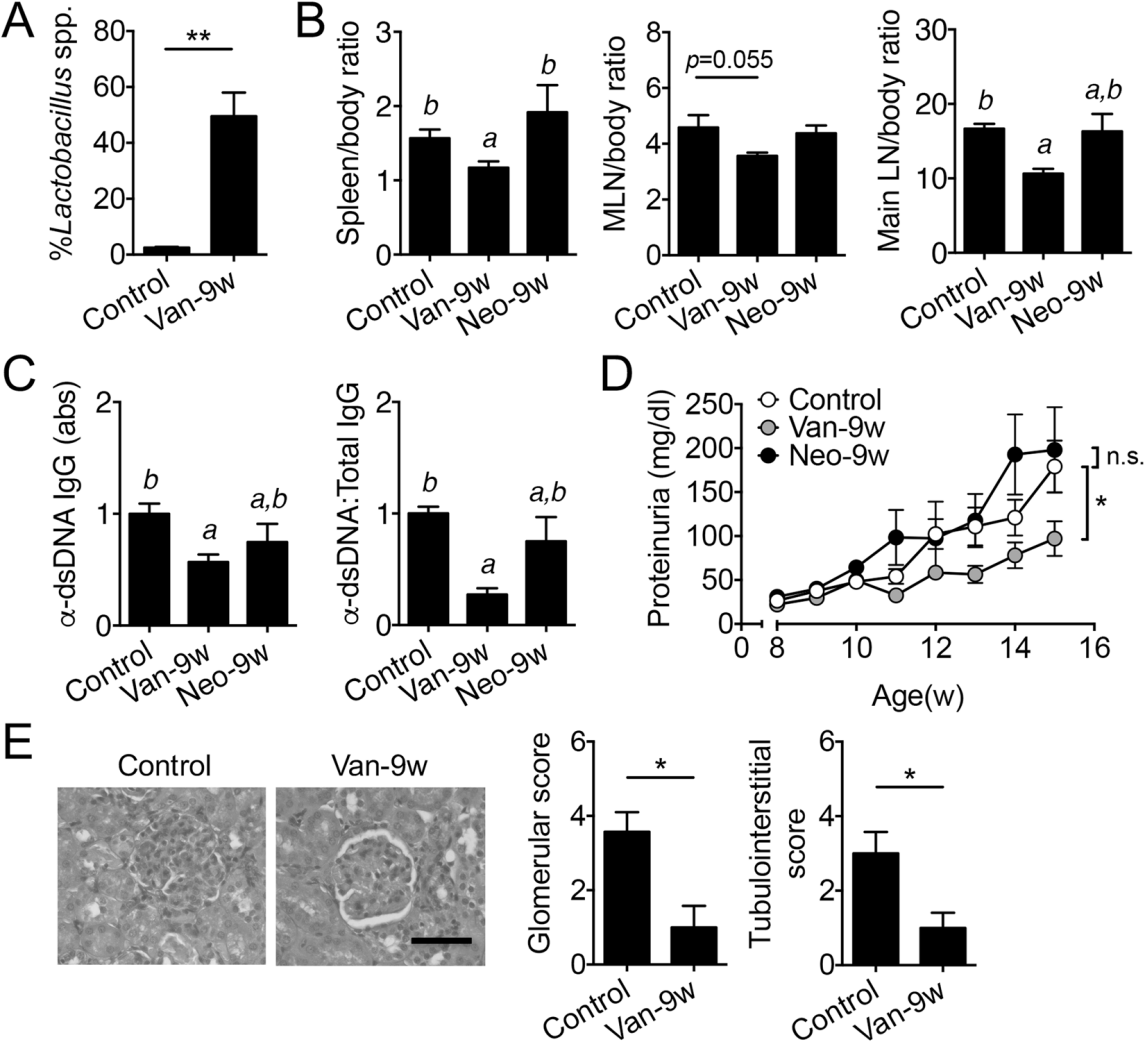
Vancomycin but not neomycin treatment started post disease onset ameliorated lupus-like disease. (**A**) Relative abundance of *Lactobacillus* spp. in the fecal microbiota at 15 weeks of age (n = 4 per group). Control: no antibiotics. Van-9w: vancomycin was given starting from 9 weeks of age. (**B**) Tissue to body weight ratio (%) for the spleen, MLN and major lymph nodes (main LN) including mesenteric, renal, inguinal, lumbar, superficial, axillary/brachial, mediastinal lymph nodes at 15 weeks of age. Neo-9w: neomycin was given starting from 9 weeks of age. (**C**) Level of anti-dsDNA IgG in the mouse serum and its ratio to total IgG at 15 weeks of age. (**D**) Level of proteinuria over time. (**E**) Renal histopathology at 15 weeks of age. Left: representative PAS-stained kidney sections; bar equals 200 µm. Middle: glomerular score. Right: tubulointerstitial score. In B-E, n = 12 in Control and Van-9w groups, n = 4 in the Neo-9w group. *p < 0.05, **p < 0.01, n.s.: not statistically significant. Letters a and b represent statistically significant difference among the groups.

A cocktail of 4 different antibiotics is impractical to implement as a treatment for lupus and more likely to induce resistance. Vancomycin alone, however, can remove Gram-positive bacteria such as Clostridial species (*Lachnospiraceae*) but spares Lactobacilli^17–20^, making it a favorable choice as a potential intervention against lupus progression in MRL/lpr mice. Importantly, vancomycin is not absorbed in the intestine^36,37^ and its effects are limited to targeting commensal bacteria in the gut lumen. We thus examined whether oral treatment of vancomycin initiated at 9 weeks of age could attenuate lupus. As anticipated, the relative abundance of *Lactobacillus* spp. was significantly elevated with vancomycin treatment (Fig. 4A). While the overall body weight did not change (Fig. S2A), vancomycin treatment significantly decreased the weight of spleen, MLN and major lymph nodes (Fig. 4B). Furthermore, vancomycin significantly reduced the level of circulating anti-dsDNA IgG (Fig. 4C), proteinuria (Fig. 4D), and renal histopathological scores (Fig. 4E). In contrast, neomycin, an antibiotic with a broad spectrum of activity against both Gram-positive and Gram-negative bacteria^38^, did not affect the severity of lupus disease when given starting from 9 weeks of age. This indicates that the decrease in bacterial load, achieved by both vancomycin and neomycin treatments, was not the reason for disease attenuation. Together, these results suggest that vancomycin given post disease onset recapitulated the attenuated disease phenotype seen with mixed antibiotic treatment.

### Vancomycin reshaped the gut microbiota and differentially affected KEGG pathways

**Figure 5:**
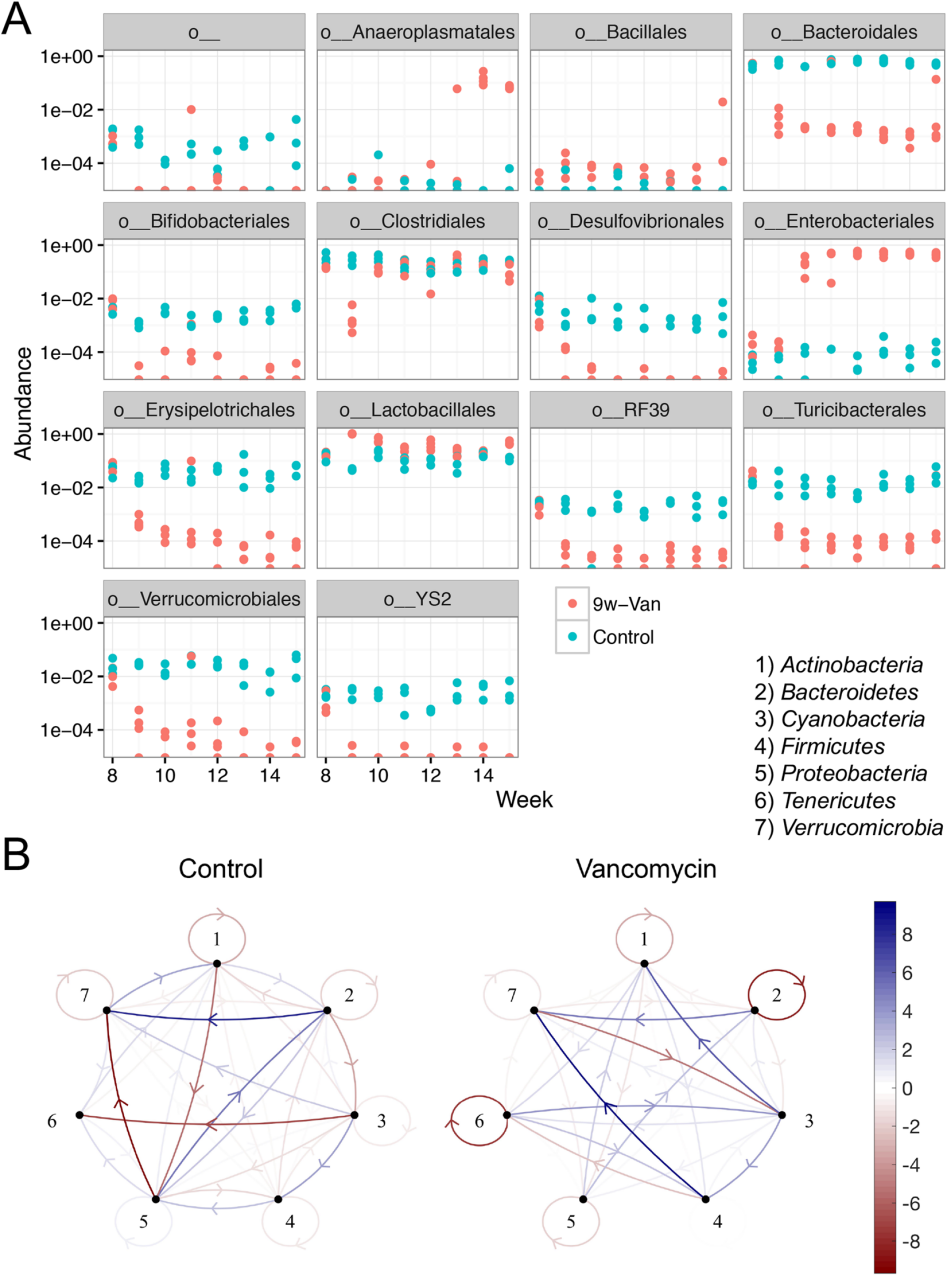
Vancomycin treatment reshaped the gut microbiota. (**A**) Time-dependent changes of the relative abundance of gut bacteria at the order level. The first label “o__” means an order with uncultured bacteria. (**B**) Mathematical networks generated based on the longitudinal changes of gut microbiota at the phylum level. 1) *Actinobacteria*, 2) *Bacteroidetes*, 3) *Cyanobacteria*, 4) *Firmicutes*, 5) *Proteobacteria*, 6) *Tenericutes*, and 7) *Verrucomicrobia*. Blue, positive influence. Red, negative influence. The thicker the line, the stronger the relationship.

We collected weekly fecal samples from vancomycin-treated mice and determined longitudinal changes of gut microbiota composition by using 16 S rRNA sequencing. The diversity of gut microbiota was largely reduced upon vancomycin administration (Fisher index, Fig. S2B). Similar to the mixed antibiotic treatment, vancomycin removed *Clostridiales* right after treatment initiation and *Bacteroidales* at most of the observed time points (Fig. 5A). Many other groups of bacteria were also removed by vancomycin, including *Desulfovibrionales* and *Turicibacterales*, whereas *Enterobacteriales* were enriched. *Anaeroplasmatales*, on the other hand, was increased by vancomycin treatment at the later time points. These results suggest that vancomycin given during active disease reshaped the gut microbiota in MRL/lpr mice.

We next established mathematical networks to model the gut microbiota changes at the phylum level (Fig. 5B). While some relationships remain the same with and without vancomycin treatment, such as the positive influence of *Bacteriodetes* on *Verrucomicrobia* (nodes 2 and 7), overall, treatment with vancomycin significantly affected the interaction among different bacterial groups. *Firmicutes* (node 4), for example, were shown to positively influence *Verrucomicrobia* (node 7) only in the vancomycin-treated group. Both *Lactobacillaceae* (“good” bacteria) and *Lachnospiraceae* (possibly “harmful” bacteria in this model) belong to the phylum *Firmicutes*. Another example is *Proteobacteria* (node 5), which are commonly used to represent Gram-negative bacteria. In untreated mice, *Proteobacteria* were involved in a complex interaction network with *Bacteroidetes* (node 2), *Actinobacteria* (node 1) and *Verrucomicrobia* (node 7), suggesting that Gram-negative bacteria may contribute to lupus pathogenesis in MRL/lpr mice. However, these interactions were absent in vancomycin-treated mice, suggesting that Gram-negative bacteria no longer play an important role to promote lupus in the presence of vancomycin.

**Figure 6:**
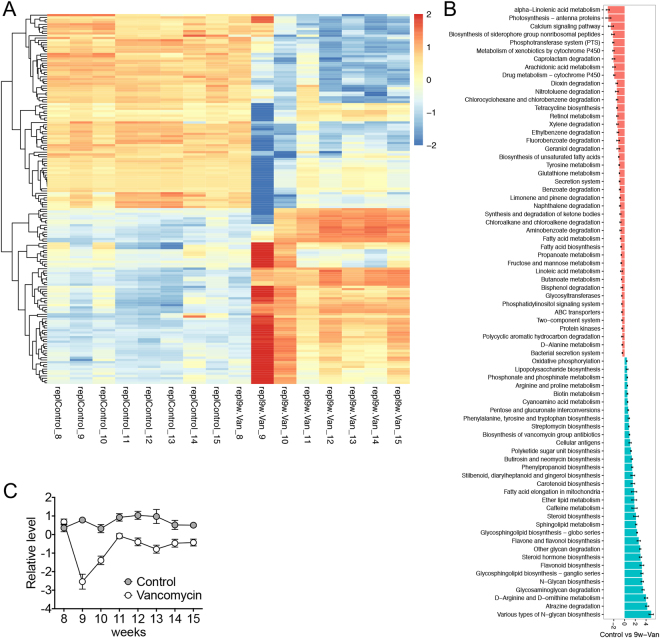
Vancomycin treatment differentially affected KEGG pathways in a time-dependent manner. (**A**) Changes of level 3 functional pathways over time. The result of PICRUSt analysis was plotted with DESeq. 2. Raw data can be found in Table S1. Note that the 9-week microbiota samples were collected 4 days after the initiation of vancomycin treatment for the Van group. (**B**) Changes of level 3 functional pathways when data from 9–15 weeks were averaged within each treatment group. 9w-Van, vancomycin was given starting from 9 weeks of age. (**C**) Changes of the average level of 12 LPS-related functional genes over time. PICRUSt analysis was performed at the ortholog level. Raw data with the names of the LPS-related functional genes can be found in Table S2.

To get insights into functional categories and pathways affected by antibiotics treatment, we performed PICRUSt analyses, and analyzed KEGG level 3 pathways with DESeq. 2. The analysis showed that the presentation of functional pathways was relatively stable over time for the control group, whereas treatment with vancomycin produced the most significant changes of the functional pathways at 9 weeks of age and 4 days after the initiation of antibiotic treatment (Fig. 6A). Some of the changes sustained beyond 9 weeks of age in the vancomycin group, while others returned to the baseline levels. Among the pathways with significant changes (Table S1), many exhibited similar trends as in our previous publication^3^. These include the vancomycin-mediated upregulation of *Peptidoglycan biosynthesis* and *Transcriptional factors* pathways that were associated with improved lupus-like symptoms in MRL/lpr mice, as well as vancomycin-mediated downregulation of *Glyoxylate and dicarboxylate metabolism*, *Histidine metabolism*, and *Phenylalanine, tyrosine and tryptophan biosynthesis* pathways that were associated with deteriorated lupus-like symptoms in MRL/lpr mice.

In addition to analysis of the time course, we also determined differential presentation of functional pathways between control and vancomycin groups when data from 9–15 weeks were averaged (Fig. 6B). This analysis showed 75 functional pathways that were significantly altered, including the *Phenylalanine, tyrosine and tryptophan biosynthesis* pathway that was associated with more severe lupus disease^3^ and significantly downregulated by vancomycin treatment. Another important pathway, *Lipopolysaccharide biosynthesis*, was also significantly downregulated by vancomycin regardless of sampling time. Detailed analysis of PICRUSt data on the ortholog level (Fig. S3 and Table S2) revealed vancomycin-mediated downregulation of 12 functional genes within the *Lipopolysaccharide biosynthesis* pathway. When the relative levels of these genes were plotted over time, they exhibited the same pattern of a sharp decrease at 9 weeks of age, followed by gradual recovery from 10–11 weeks of age (Fig. 6C). Importantly, a majority of these genes were *Lpx* genes involved in lipid A biosynthesis^39^. Lipid A is the endotoxic component of LPS. These analyses suggest that vancomycin may attenuate lupus-like disease in MRL/lpr mice by downregulating the relative abundance of Gram-negative bacteria and the LPS endotoxin.

### Vancomycin decreased intestinal permeability and the plasma level of LPS

**Figure 7:**
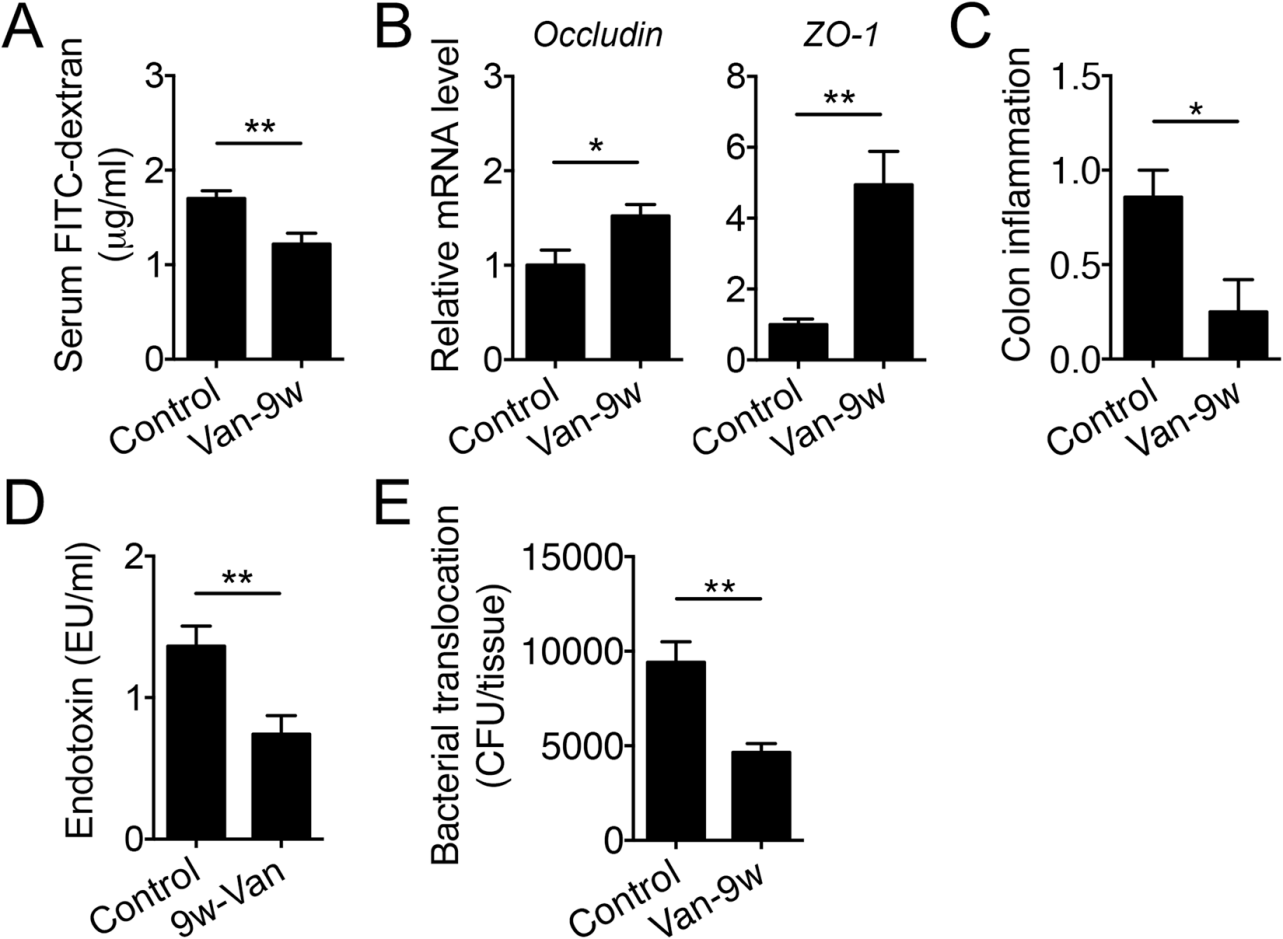
Vancomycin treatment decreases intestinal permeability and the serum level of LPS. (**A**) Diffusion of FITC-conjugated dextran into the circulation after oral gavage as a direct measurement of intestinal permeability (n ≥ 8 per group). Mice were at 15 weeks of age when the assay was performed. (**B**) Transcript levels of barrier-forming tight junction proteins in the intestinal epithelium in 15-week-old MRL/lpr mice (n = 4 per group). (**C**) Inflammation in the colon at 15 weeks of age as determined by histopathological scoring (n ≥ 6 per group). (**D**) Serum level of LPS in 15-week-old MRL/lpr mice (n ≥ 12 per group). (**E**) Bacterial translocation to the MLN at 15 weeks of age (n ≥ 7 per group). Bacterial CFU per tissue is shown. *p < 0.05, **p < 0.01.

LPS accelerates lupus progression in several lupus-prone mouse models^21–25^. A leaky gut may allow for the translocation of Gram-negative bacteria across the intestinal epithelium, leading to an increase of LPS—a cell wall component of Gram-negative bacteria—in the circulation. We thus determined the intestinal permeability of vancomycin-treated mice by measuring the diffusion of orally gavaged (for small intestine) or rectally administered (for colon) FITC-conjugated dextran. The result showed that vancomycin treatment significantly decreased the permeability of small intestine (Fig. 7A) and had a trend to decrease colonic permeability (Fig. S4A). Neomycin, on the other hand, did not change intestinal permeability (Fig. S4B). In addition, vancomycin significantly increased the epithelial expression of barrier-forming tight junction transcripts *Occludin*, *ZO-1* (Fig. 7B), *Cldn1* and *Cldn3* (Fig. S4C), whereas the transcript level of pore-forming tight junction protein Cldn2 did not change with vancomycin treatment (data now shown). While the intestinal epithelium appeared intact regardless of treatment, significantly less inflammation was observed with vancomycin treatment (Fig. 7C). Further studies that directly measured LPS in the circulation indicated that vancomycin indeed significantly decreased the serum level of LPS (Fig. 7D). This is consistent with significantly reduced bacterial translocation to MLN with vancomycin treatment (Fig. 7E). Together, these results suggest that vancomycin may attenuate lupus-like disease in MRL/lpr mice by reducing the “leakiness” of the gut epithelium and preventing the translocation of LPS and/or LPS-containing bacteria from the gut lumen to the circulation.

## Discussion

In previous studies we have found that the gut microbiota of lupus-prone MRL/lpr mice contain “good” and “bad” commensal bacteria that may attenuate or facilitate disease progression, respectively^3^. Complete removal of gut microbiota, achieved by GF housing, does not affect the disease outcome as both “good” and “bad” bacteria are removed leading to neutralization of their respective effects. Consistent with this, our preliminary observations suggest that antibiotic treatment initiated from 3 weeks of age (the time of weaning) till the endpoint does not influence disease activity in MRL/lpr mice (unpublished results). Antibiotic treatment started post disease onset, on the other hand, appear to target “bad” bacteria (*Lachnospiraceae*) for removal while enriching “good” bacteria (*Lactobacillus* spp.), thereby attenuating lupus. Our observations are highly relevant to human SLE, as a similar and significant increase of *Lachnospiraceae* was also found when comparing the feces of SLE patients to those of healthy individuals, whereas Lactobacilli were not detectable in either group of people (unpublished results). This suggests that an appropriately selected antibiotic may attenuate disease flares in SLE patients by targeting *Lachnospiraceae* for removal.

Our results have shown that a single antibiotic vancomycin can recapitulate the beneficial effect of mixed antibiotics (ampicillin, vancomycin, neomycin and metronidazole) against lupus progression in MRL/lpr mice. Interestingly, while both treatments reduced the bacterial load, vancomycin was able to decrease the bacterial diversity but the mixed antibiotics were not. Increased gut bacterial diversity is associated with more severe lupus in mice^3^ whereas decreasing bacterial diversity with acidified water attenuated lupus^10^. Mixed antibiotics have been shown to decrease gut bacterial diversity in other mouse models^40,41^, but the microbiota community structure can be resilient to antibiotic treatment for days or even weeks^42^. It is currently unclear why the mixed antibiotics did not reduce the bacterial diversity in MRL/lpr mice when given from 9–15 weeks of age, but the antibiotics were initiated after the microbiota had been established, and that a lower dose of each antibiotic (0.5–1 g/l) than vancomycin alone (2 g/l) was used. In future investigations, we will perform dose response experiments to elucidate the relationships between the dose of antibiotics, gut bacterial diversity and the severity of lupus disease.

Vancomycin is known to remove Gram-positive bacteria but spares Lactobacilli. In the current study, when the lupus-prone MRL/lpr mice were treated with vancomycin, their gut microbiota also showed a marked increase in the relative abundance of Lactobacilli. With the reshaped gut microbiota, the intestinal permeability also decreased. This may be due to the upregulation of tight junction proteins in the intestinal epithelium, which are responsible for blocking the paracellular passages between the intestinal epithelial cells^43^. These results are consistent with a recent report showing that vancomycin, rather than amoxicillin and metronidazole, results in less permeable intestines^44^. The increased abundance of Lactobacilli might be the mechanism by which vancomycin enhanced the intestinal barrier function. Numerous *Lactobacillus* strains, such as *L. rhamnosus* and *L. reuteri*, have been reported to improve the gut barrier function^45,46^.

Vancomycin treatment significantly reduced the level of LPS/endotoxin in the circulation. This observation correlates well with our mathematical modeling result that *Proteobacteria* (representing Gram-negative bacteria), while influencing the relative abundance of *Bacteroidetes*, *Actinobacteria* and *Verrucomicrobia* in control mice, were no longer able to affect these bacteria in vancomycin-treated mice. In addition, the decrease in circulating LPS is also consistent with the result of our PICRUSt analysis where the *Lipopolysaccharide biosynthesis* pathway was significantly downregulated by vancomycin treatment. Interestingly, however, vancomycin did not significantly reduce the relative abundance of LPS-containing Gram-negative bacteria (*Proteobacteria*). Within the phylum *Proteobacteria*, the order *Enterobacteriales* was significantly upregulated, whereas the order *Desulfovibrionales* was significantly downregulated, by vancomycin treatment. This led to an averaged effect of no change in the relative abundance of *Proteobacteria* between control and vancomycin-treated groups. Importantly, bacteria in the order *Desulfovibrionales* are resistant to vancomycin^47^, suggesting that the downregulation of *Desulfovibrionales* may be due to their interaction or symbiotic relationship with vancomycin-sensitive Gram-positive bacteria. Moreover, it is speculated that the decrease in *Desulfovibrionales* might be due to reduced proliferation of the bacteria rather than cell death—as they are resistant to vancomycin-mediated killing—so that free LPS is not released to the circulation. The reduction of circulating LPS with vancomycin treatment, on the other hand, could be due to increased intestinal barrier function and reduced translocation of Gram-negative bacteria, such as those in the order *Enterobacteriales*, from the gut lumen to the circulation.

Increased intestinal permeability, or a leaky gut, has arisen in recent years as one contributing factor for autoimmune disease^48,49^. Studies of type 1 diabetes have provided strong evidences to support this notion^50,51^. In SLE, however, the investigation on the interaction between the leaky gut and disease development is still in its infancy. We have previously shown that MRL/lpr mice, compared to age-matched healthy controls, exhibit a higher serum level of LPS before disease onset (unpublished data). Here, the beneficial effects of vancomycin treatment are accompanied by a reversal of the leaky gut and likely less translocation of LPS across the intestinal epithelium. Although the effects were not as significant, the mixed antibiotic treatment also reversed the leaky gut by decreasing the intestinal permeability (Fig. S4D) and increasing the expression of barrier-forming tight junction transcripts (Fig. S4E). Together, these results suggest that a leaky gut may drive the initiation and/or progression of lupus disease at least in mice. It is noteworthy that SLE-like disease in mice is characterized by inflammation of specific organs such as the kidney, and this may not be a systemic response as seen in human SLE.

In the past decades, SLE occurrence has increased several folds in the developed world^2^. The Western diet—high in fat but low in fiber—could have contributed to this by affecting the gut microbiota^52–54^. Notably, the gut leakiness and microbial translocation are also involved in this process^54^. Future investigations are necessary to study the interactions among the leaky gut, microbial translocation and correspondingly the aggravated chronic diseases, such as SLE. At the same time, factors that can reverse gut leakiness, including probiotics and polyunsaturated fatty acids^55,56^, should be considered as part of the disease management strategies.

The cause of lupus is unclear and there is no known cure. Current treatments for SLE are primarily nonselective immunosuppressants. They can effectively treat symptoms, but the side effects are a major cause of concern. Patients taking long-term immunosuppressants are prone to higher incidence of and more severe infections. There is an imperative need for new treatment strategies against SLE, for which a better understanding of disease pathogenesis is required. The results of this study showed that antibiotic treatment given after disease onset in MRL/lpr mice ameliorated lupus-like symptoms, reducing the size of lymphoid organs, decreasing the level of circulating autoantibodies, and attenuating lupus nephritis. The decrease in disease activity was accompanied by decreases in various IL-17-producing cells and an increase of circulating IL-10. In addition, antibiotic treatment reshaped the composition of the gut microbiota, increasing the relative abundance of *Lactobacillus* spp. (“good” bacteria) while decreasing that of *Lachnospiraceae* (“bad” bacteria). Importantly, the therapeutic benefit of the mixed antibiotic treatment could be recapitulated by a single antibiotic vancomycin, which also favored *Lactobacillus* spp. Detailed analyses of the microbiota through mathematical and functional approaches indicate that *Proteobacteria*, or Gram-negative bacteria, and/or their structural component LPS may contribute to lupus progression in MRL/lpr mice. Further studies revealed that vancomycin reduced intestinal permeability and decreased the translocation of LPS and/or LPS-containing gut microbiota through the intestinal epithelium, thus preventing LPS from accelerating lupus disease. Taken together, these results suggest that antibiotics, especially vancomycin, may be beneficial as a treatment for lupus through reshaping the gut microbiota.

## Methods

### Mice and antibiotic treatment

MRL/lpr mice (stock #000485) were purchased from The Jackson Laboratory (Bar Harbor, ME) and maintained in a specific pathogen-free facility. All mice used were female as lupus has a strong female bias. Antibiotic mixture (1 g/l ampicillin, 1 g/l neomycin, 1 g/l metronidazole and 0.5 g/l vancomycin) was given in the drinking water starting from 9 weeks of age till euthanasia at 16 weeks of age. For single antibiotic treatment, 2 g/l vancomycin or 2 g/l neomycin was given in the drinking water from 9 weeks of age till euthanasia at 15 weeks of age. The drinking water with antibiotics was refreshed every 5 days. This study was carried out in strict accordance with the recommendations in the Guide for the Care and Use of Laboratory Animals of the National Institutes of Health. The protocol was approved by the Institutional Animal Care and Use Committee (IACUC) of Virginia Tech College of Veterinary Medicine (Animal Welfare Assurance Number: A3208-01). For anesthesia and euthanasia, isoflurane and CO_2_ were used, respectively, according to the IACUC protocol. All experiments were performed in accordance with relevant guidelines and regulations.

### Microbiota 16S and PICRUSt inferred metagenomics analyses

Fecal microbiota samples were obtained by taking an individual mouse out of the cage and collecting a fecal pellet. The 9-week fecal pellet was taken 4 days after the initiation of antibiotic treatment. To avoid cross-contamination, each microbiota sample was collected by using a new pair of sterile tweezers. All samples were stored at −80 °C till being processed at the same time. Sample homogenization, cell lysis and DNA extraction were performed as previously described^3,57^. The V4 region of purified 16 S rRNA gene amplicons were sequenced bi-directionally (paired-end 150 bp) on an Illumina MiSeq. Microbiome data analysis was performed as previously described^3,57^. Briefly, OTUs were picked by usearch, taxonomy was assigned against Greengenes reference database. Microbial diversity measures including Fisher and observed species were calculated by using QIIME. The datasets generated and analyzed during the current study are available in the NCBI number SRP102626.

Bacterial metagenomes were predicted using PICRUSt^58^ by comparing 16 S to database gg13.5 then to Integrated Microbial Genomes (IMG). The OTUs were mapped to gg13.5 database at 97% similarity by QIIME’s “pick_closed_otus” script. The OTUs abundance was normalized using 16 S rRNA gene copy numbers from known bacterial genomes. The normalized OTUs were used for metagenomes prediction in PICRUSt. The predicted Kyoto Encyclopedia of Genes and Genomes (KEGG) orthologs^59^ was summarized to level-3 functional categories and compared among groups by using the Statistical Analysis of Metagenomic Profile package^60^. Differentially represented gene families were identified by two-sided Welch’s *t*-test with Storey’s false-discovery-rate correction. DESeq. 2 analysis^61^ in R environment was performed with read count data for statistical analysis [R 2016, version 3.3.2 https://www.r-project.org/]. We compared between treatments and control for each week (vancomycin *vs*. control at 8 week, 9 week, etc.). We also compared consecutive weeks for each genotype respectively (e.g. 8 week *vs*. 9 week for vancomcyin), and a time course analysis which tests interaction between time and treatment. Functional categories were considered significantly differentially abundant between conditions if their adjusted *P* value was ≤ 0.001 and their absolute value of log_2_ fold change was ≥ 2. Categories with low average count were filtered before the analysis. Read counts for each KEGG category were normalized by DESeq. 2 and averaged between replicates. The average read counts were centered and scaled before used for clustering analysis to produce a heatmap.

### Mathematical modeling

A standard way in mathematically describing the temporal dynamics of interacting species is the generalized Lotka-Volterra or predator-prey system, which reads as follow

1 $${\boldsymbol{y}}^{\prime} ({t})=\mathrm{diag}({\bf{y}}({t}))({\bf{r}}+{\bf{Ay}}({t})),$$

where (**y**(*t*)) is the diagonal matrix with the state variable **y**(*t*) = [*y*_1_(*t*), …, *y*_*n*_(*t*)]^T^ (describing the temporal abundances of the different organisms), **r** is the vector of intrinsic growth rate parameters, and **A** is the interaction matrix that characterizes the influences within the network of each species, e.g., the entry *a*_*ij*_ of the matrix **A** describes the influence of species *j* on the growth of species *i* [M. Chung, J. Krueger, and Mihai Pop. Robust Parameter Estimation for Biological Systems: A Study on the Dynamics of Microbial Communities. In revision at *Mathematical Bioscience*, 2017. [arXiv Preprint]]. Hence, the network of the species is identified by this interaction matrix **A**.

To determine the interaction **A** we assumed that the dynamics were at an asymptotically stable equilibrium $\overline{{\boldsymbol{y}}}$, where $\overline{{\boldsymbol{y}}}$ was determined by the data (Fig. 5A). Mathematically such equilibria are given by solution of the nonlinear equation

2 $$\begin{array}{c}\mathrm{diag}(\overline{{\bf{y}}})({\bf{r}}+{\bf{A}}\overline{{\bf{y}}})={\bf{0}}\\ {\rm{subject}}\,{\rm{to}}\,{\rm{the}}\,{\rm{constraint}}\,{{\rm{\max }}}_{j}\Re e({\lambda }_{j}) < 0,\end{array}$$

where ℜℯ and is the real part of *λ*_*j*_ and *λ*_*j*_ is the *j* ^th^ eigenvalue of the matrix diag(**r**+**A** $\overline{{\boldsymbol{y}}}$) + diag($\overline{{\boldsymbol{y}}}$)**A**.

Hence, we need to find **A** such that the above equations are fulfilled. Note that this is a nonconvex problem and multiple solution may exist. More precise, multiple network configuration may describe the same asymptotically stable equilibrium $\overline{{\boldsymbol{y}}}$. We used a repeated Monte Carlo sampled direct search method [Wright, Stephen and Nocedal, Jorge, Numerical Optimization, Springer, 2006] to identify solution **A** of above problem, see Fig. 5B.

### Renal function

Urine samples were collected biweekly and all samples were stored at −20 °C till being processed at the same time. We used Pierce Coomassie Protein Assay Kit (Thermo Scientific) to test the total protein level in the mouse urine. When mice were euthanized at 14 weeks of age, kidneys were fixed in formalin for 24 h, paraffin embedded, sectioned, and stained with Periodic acid–Schiff (PAS) at the Histopathology Laboratory at Virginia Maryland Regional College of Veterinary Medicine. Slides were read with an Olympus BX43 microscope. All slides were scored in a blinded fashion by a certified veterinary pathologist (Cecere). Glomerular lesions were graded on a scale of 0 to 3 for each of the following five categories: increased cellularity, increased mesangial matrix, necrosis, the percentage of sclerotic glomeruli, and the presence of crescents. Similarly, tubulointerstitial lesions were graded on a scale of 0 to 3 for interstitial mononuclear infiltration, tubular damage, interstitial fibrosis, and vasculitis.

### Endotoxin quantification and enzyme-linked immunosorbent assays (ELISA)

Separated serum after blood clotting was saved at −20 °C until use. We used Pierce LAL Chromogenic Endotoxin Quantitation Kit (Thermo Scientific) to measure serum endotoxin level by following the kit’s instructions. For detection of anti-double-stranded DNA (dsDNA) IgG, we used previously described methods^26^. Serum IgG, IL-6 and IL-10 concentrations were determined with mouse IgG (Bethyl Laboratories), IL-6 (Biolegend) and IL-10 (Biolegend) ELISA kits according to the manufacturer’s instructions.

### Intestinal permeability and colonic histopathology

*In vivo* intestinal permeability assay to assess barrier function was performed using Fluorescein isothiocyanate conjugated dextran (FITC-dextran, Sigma-Aldrich) method. For permeability of the small intestine, mice were water starved overnight then orally gavaged with FITC-dextran (40 mg/100 g body weight). For colonic permeability, FITC-dextran (20 mg/100 g body weight) were rectally administered twice with a 2-h break. After 4 h (for colonic permeability, 4 h after initial administration), mice were anesthetized and blood was collected and saved in the dark. Serum was then prepared from the blood, diluted 1:1 with PBS, and measured in a 96-well microplate in duplicates for the concentration of FITC in the serum by Glomax (Promega) with an excitation wavelength of 485 nm and an emission wavelength of 528 nm, using serially diluted FITC-dextran as the standards. Colonic histopathology was graded on a scale of 0 to 4 for each of the following six categories: inflammation, edema, epithelial defects, crypt atrophy, hyperplasia, and dysplasia^62^. Only inflammatory histopathology was observed in our samples. All slides were scored in a blinded fashion (Eden).

### Cell isolation and flow cytometry

Spleen, MLN and kidney were collected and mashed in 70-μm cell strainers with C10 media (RPMI 1640, 10% fetal bovine serum, 1 mM sodium pyruvate, 1% 100 MEM non-essential amino acids, 10 mM HEPES, 55 μM 2-mercaptoethanol, 2 mM L-glutamine, 100 U/ml penicillin-streptomycin, all from Life Technologies, Grand Island, NY). For splenocytes, red blood cells were lysed with RBC lysis buffer (eBioscience, San Diego, CA). To isolate intestinal epithelial cells (IECs), the entire intestine including small intestine and colon was opened longitudinally and cut into pieces. The pieces were incubated twice in EDTA-DTT solution and intensively vortexed to harvest IEC-enriched fractions. For surface marker staining, cells were blocked with anti-mouse CD16/32 (eBioscience), stained with fluorochrome-conjugated antibodies, and analyzed with Attune NxT flow cytometer (Thermo Scientific). For intracellular staining, Foxp3 Fixation/Permeabilization kit (eBioscience) was used. Anti-mouse antibodies used in this study include: CD3-APC-eFluor 780, CD8-PE-Cy7, CD4-PerCP-Cy5.5, RORγT-PE, CD3e-biotin (eBioscience); CD45-APC-Cy7, IL-10-BV421, IL-17A-APC, CD49b-biotin, CD19-biotin (Biolegend, San Diego, CA); Biotin-FITC (MACS). Flow cytometry data were analyzed with FlowJo.

### Reverse transcription-quantitative polymerase chain reaction (RT-qPCR)

Isolated IECs were homogenized with Bullet Blender homogenizer (Next Advance, Averill Park, NY) and total RNA was extracted with RNeasy Plus Mini Kit (Qiagen, Valencia, CA) according to the manufacturers’ instructions. Genomic DNA was removed by digestion with RNase-free DNase I (Qiagen). Reverse transcription was performed by using iScript cDNA Synthesis Kit (Bio-Rad, Hercules, CA). Quantitative PCR was performed with iTaq Universal SYBR Green Supermix (Bio-Rad) and ABI 7500 Fast Real-Time PCR System (Applied Biosystems, Grand Island, NY). Relative quantities were calculated using *Villin* as the housekeeping gene. Primer sequences for mouse *Villin, ZO-1, Occludin, Cldn1 and Cldn3* are available upon request.

### Tissue extraction for ELISA

Total protein was extracted from spleen according to a published method^63^ for the measurement of IL-17 with ELISA. Briefly, frozen splenic tissue was homogenized in ice-cold extraction buffer containing protease inhibitor mixture (Sigma-Aldrich). After homogenization, samples were maintained at 4 °C for 2 h, followed by centrifugation at 12,000×*g* for 20 min. Supernatants were collected and analyzed for IL-17A with ELISA (Biolegend).

### Measurement of bacterial translocation

MLN was homogenized in PBS at 0.1 g tissue/ml PBS. After short spin-down, 100 μl supernatant was added to a blood agar plate (Hardy Diagnostics) and cultured at 37 °C for 36 h. The number of colonies was counted and calculated as colony-forming units (CFU) per g tissue.

### Statistical analysis

Statistical analyses were performed by using R version 3.0.2 (sequencing data) or Prism GraphPad (non-sequencing data). Principal coordinate analysis was tested for significance by a permutational multivariate method PERMANOVA^64^. Analysis of non-sequencing data was performed with nonparametric Mann-Whitney test for the comparison of 2 groups, and nonparametric Kruskal-Wallis test for the comparison of 3 groups. The results were considered statistically significant when *p* < 0.05.

## Electronic supplementary material

Supplemental figures

Table S1

Table S2
