# Supplementary material for: Antibiotics ameliorate lupus-like symptoms in mice
Source: Sci Rep. 2017 Oct 20;7:13675. doi: 10.1038/s41598-017-14223-0 (PMC5651817; doi:10.1038/s41598-017-14223-0)
Supplement: Supplementary file 1 — Supplemental figures [file 41598_2017_14223_MOESM1_ESM.pdf]

Antibiotics ameliorate lupus-like symptoms in mice

Qinghui Mu, Vincent J. Tavella, Jay L. Kirby, Thomas E. Cecere, Matthias Chung, Jiyoung Lee, Song Li, S. Ansar Ahmed, Kristin Eden, Irving C. Allen, Christopher M. Reilly, Xin M. Luo

Supplemental Figure 1

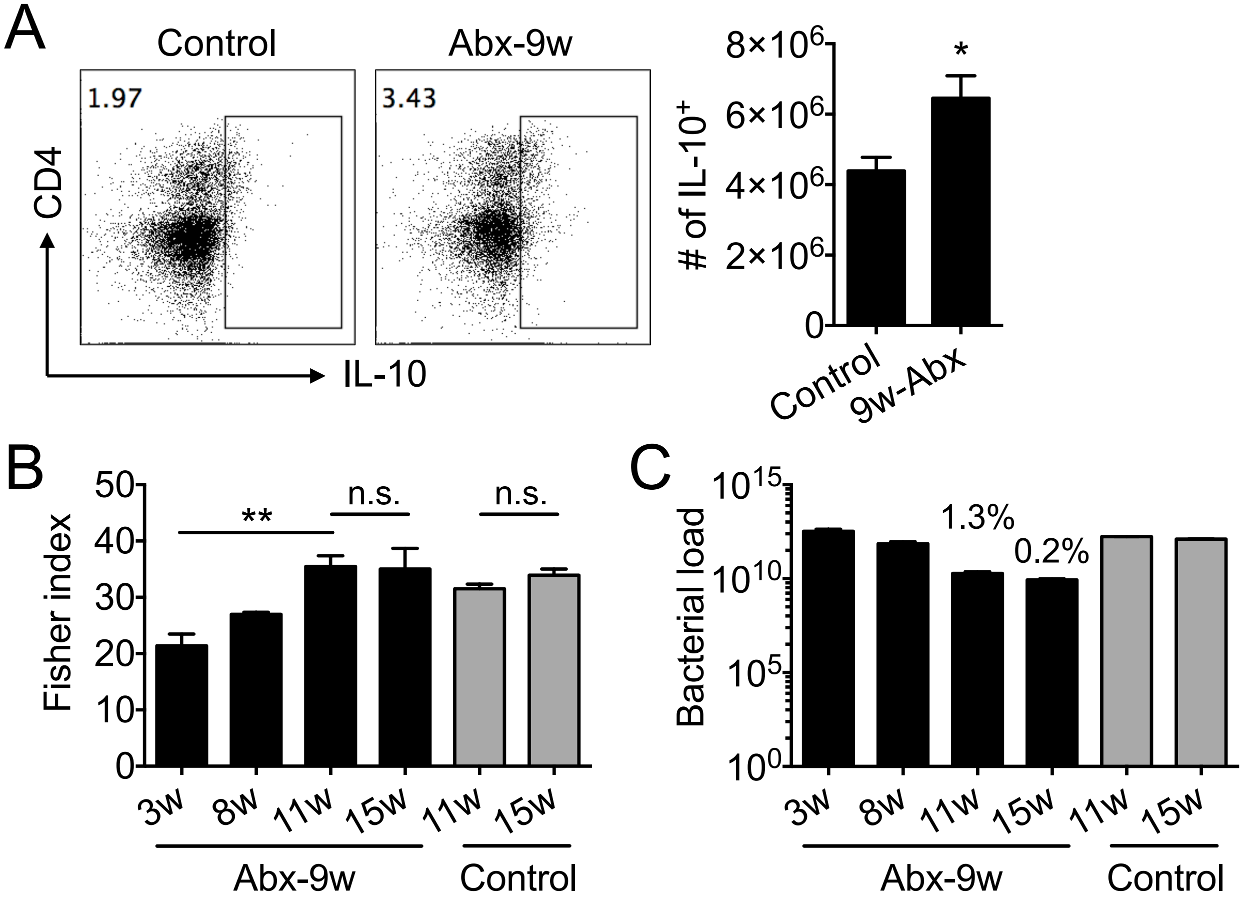

Supplemental Figure 1. (**A**) Percentage and number of IL-10-producing cells in the MLN. (**B**) Bacterial diversity upon antibiotic treatment. Fisher index is shown. (**C**) Bacterial load upon antibiotic treatment. The numbers shown are percentage of bacteria left in the gut microbiota. $*p<0.05$, $**p<0.01$. n.s., not significant.

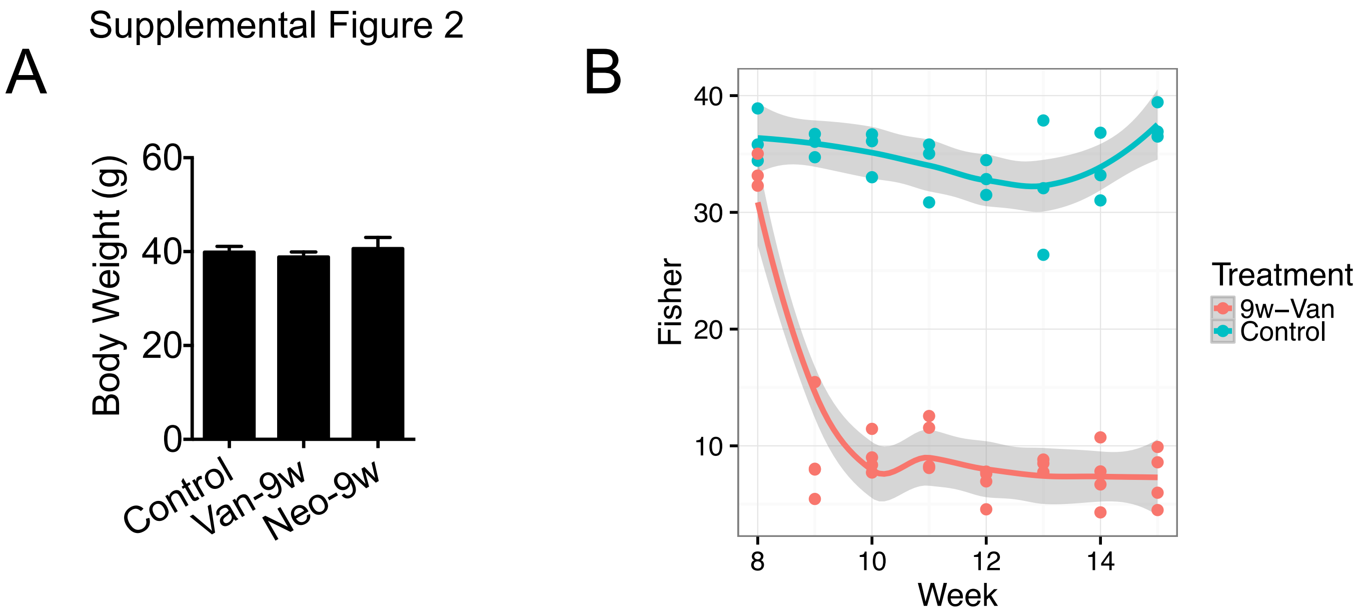

Supplemental Figure 2. (**A**) Body weight of vancomycin- or neomycin-treated mice. (**B**) Bacterial diversity upon vancomycin treatment. Fisher index is shown.

Supplemental Figure 3

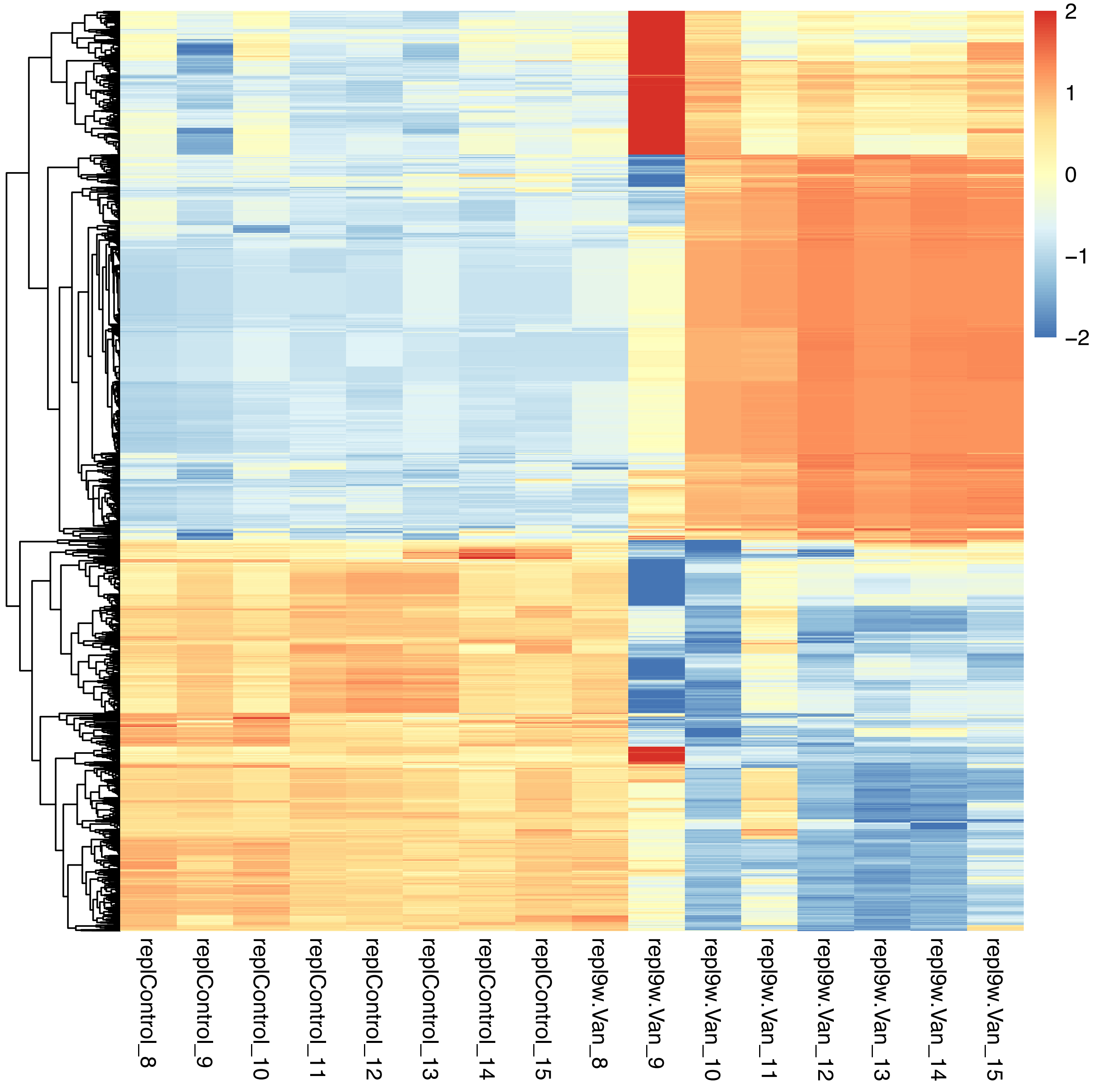

Supplemental Figure 3. KEGG analysis (PICRUSt) at the ortholog level. The raw data are shown in Table S2.

Supplemental Figure 4

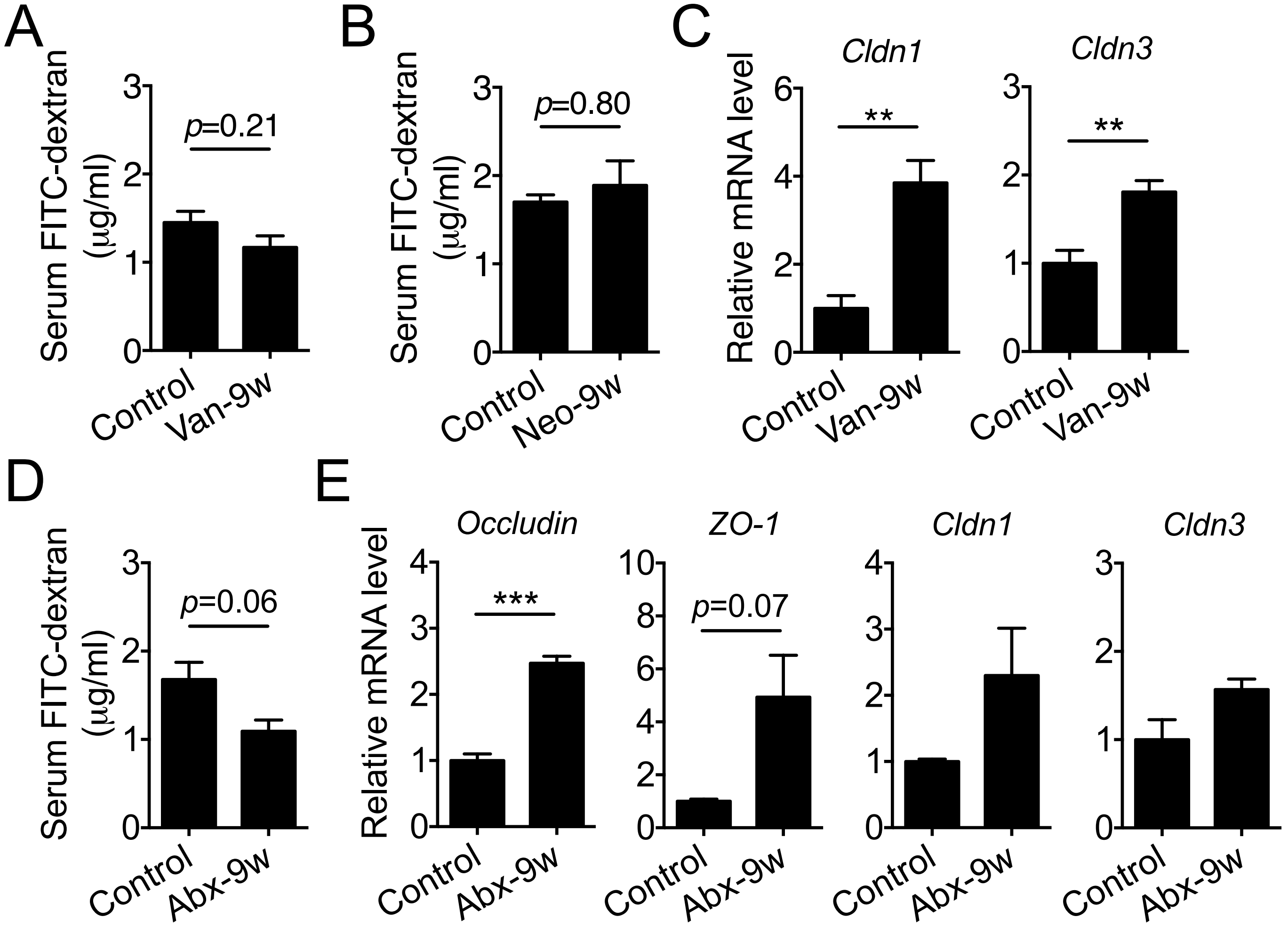

Supplemental Figure 4. (**A**) Colonic permeability as determined by FITC-dextran diffusion assay. (**B**) Diffusion of oral FITC-dextran to the blood upon neomycin treatment. (**C**) Relative transcript levels of Cldn1 and Cldn3 in IECs upon vancomycin treatment. (**D**) Diffusion of oral FITC-dextran to the blood upon mixed antibiotic treatment. (**E**) Relative transcript levels of Occludin, ZO-1, Cldn1 and Cldn2 in IECs upon mixed antibiotic treatment. **p<0.01, ***p<0.001. All measurements were done when mice were 15 weeks of age.
